# Narcissism predicts noise perception but not signal decoding in emotion

**DOI:** 10.1038/s41598-023-41792-0

**Published:** 2023-09-02

**Authors:** Anna Z. Czarna, Heidi Mauersberger, Till Kastendieck, Roksana R. Zdunek, Constantine Sedikides, Ursula Hess

**Affiliations:** 1 Institute of Applied Psychology, Jagiellonian University, Kraków, Poland; 2 Humboldt-Universität Zu Berlin, Berlin, Germany; 3 School of Psychology, University of Southampton, Southampton, UK

**Keywords:** Human behaviour, Personality

## Abstract

Grandiose narcissists claim that they have better-than-average emotion recognition abilities, but many objective tests do not support this claim. We sought to clarify the relation between grandiose (both agentic and communal) narcissism and emotion recognition by taking a closer look at the components of emotion recognition. In two studies (*N*_1_ = 147, *N*_2_ = 520), using culturally distinct samples and different stimulus materials, we investigated the relation between grandiose narcissism and signal decoding (accurate view of the intended emotion displayed in an expression) as well as noise perception (inaccurate deciphering of secondary emotions that are not part of the emotional message). Narcissism was inconsistently related to signal decoding, but consistently and positively related to noise perception. High grandiose (agentic and communal) narcissists are not necessarily better at signal decoding, but are more susceptible to noise perception. We discuss implications for narcissists’ social interactions and interpersonal relationships.

## Introduction

The item “I can read people like a book” in the Narcissistic Personality Inventory (NPI)^1^, a popular scale of grandiose agentic narcissism, is indicative of high levels of that trait. Indeed, grandiose agentic narcissists report better-than-average emotion recognition abilities^2,3^. However, objective tests of emotion recognition have yielded mixed findings. In this article, we sought to clarify the relation between grandiose narcissism—both agentic and communal—and emotion recognition by breaking down the latter construct into two components: (a) signal decoding, that is, accurate decoding of the intended emotion displayed in an expression, and (b) noise perception, that is, inaccurately discerning secondary emotions in the emotional display that are not part of the emotional message^4^. We report two studies that we conducted in different cultures (Germany, Poland) using different stimulus materials.

Narcissism is a multifaceted construct, normally distributed in the population. Individuals high in narcissism (often referred to as “narcissists”) believe that they are special, superior, and entitled, while treating others unempathetically and often antipathetically^5^. The common thread among narcissism’s various forms is egocentric exceptionalism and social selfishness.

The most extensively researched form of narcissism is grandiose narcissism, which is characterized by an extraverted, exhibitionistic, self-assured, dominant, and manipulative interpersonal orientation^5^. Adopting a more granular approach, we examined two forms of grandiose narcissism, agentic and communal^6,7^. Agentic narcissists self-enhance (i.e., inflate their self-views) on the agentic domain (e.g., competence, creativity, intelligence, scholastic aptitude), whereas communal narcissists self-enhance on the communal domain (e.g., warmth, prosociality, morality, interpersonal skills). We use the term agentic narcissism to refer specifically to agentic narcissism as conceptualized in the circumplex framework of agentic versus communal grandiose narcissism and assessed using the NPI. This should not be mistaken for “agentic narcissism” as defined by the Five-Factor Model’s distinction between agentic and antagonistic grandiose narcissism, or as assessed by the Five-Factor Narcissism Inventory^8^. Whereas agentic narcissists consider themselves particularly capable, knowledgeable, and driven, communal narcissists consider themselves overly altruistic or fair, claim that they trust others, and react with moral indignation at perceived unfairness. Yet, in the long-run neither form of narcissism is well-liked by peers^9,10^. One reason might be narcissists’ incompetence in interpreting the emotional expressions of their interaction partners, leading to awkward and uncomfortable encounters.

People vary in their emotion recognition ability, that is, the degree to which they are able to decode others’ emotions. A burgeoning literature (20 studies, in total) has examined the relation between agentic narcissism and emotion recognition (Supplementary Table S1). The results are inconsistent. Eight studies found no relation between the two variables. Nine studies found that high (vs. low) agentic narcissists were impaired in emotion recognition: They recognized emotions expressed at lower intensities less accurately, required more information to recognize emotions, or were slower to recognize emotions. Lastly, five studies found that high (vs. low) narcissists were better at recognizing certain emotions.

An examination of the findings reveals multiple sources of inconsistency. One set of studies reported that high (vs. low) agentic narcissists were less likely to recognize distinct emotions such as fear^11,12^, hatred (but not anger)^13^, or sadness and surprise^12^. Another set of studies reported that high (vs. low) agentic narcissists were more likely to recognize distinct emotions such as anger^3^. These studies used a variety of standardized emotion recognition tests (Supplementary Table S1). However, even studies employing the same instruments (i.e., Reading the Mind in the Eyes Test or RMET)^14^ yielded discrepant results: from superior^15,16^ through no different^17–19^ to inferior^20,21^ emotion recognition by high (vs. low) agentic narcissists.

The above-reviewed studies differ in statistical power, sample characteristics, scales used to assess narcissism, as well as emotion recognition tests, and these differences might be partly responsible for the heterogeneity of results. Still, even properly powered studies on samples with similar characteristics, involving standard narcissism assessment instruments (e.g., the NPI) and emotion recognition tests (e.g., the RMET), produced discrepant results^16,17,21^. Thus, despite considerable empirical effort, one cannot draw straightforward conclusions about narcissism and emotion recognition. We suggest an explanation: None of the studies addressed separately signal decoding and noise perception in emotion recognition.

The studies on narcissists’ emotion recognition have used standardized tests or stimuli (Supplementary Table S1). These tests instruct participants to interpret a given expression by selecting from a list of labels the focal emotion, that is, the emotion label that best describes a given facial expression. The participant makes an accurate judgment if they select the correct emotion label. Performance-based tests of emotion recognition are usually scored based on consensus. Here, “accurate recognition” means decoding the emotion that the expresser reported they felt and/or that the majority of perceivers recognized in the expression. A given judgment, then, is either accurate or inaccurate. Overall emotion recognition accuracy is calculated based on hit rates, that is, the proportion of target stimuli correctly identified. However, this approach does not reflect the full range of emotion recognition in daily life^22^. Indeed, people often perceive emotions as mixed or blended, even when these are presented in the form of an emotion prototype^23^. Α perceiver may correctly infer the intended emotion displayed in the expression (i.e., signal), but also infer additional and secondary emotions that are not part of the expression (i.e., noise). The additional, secondary emotions represent not necessarily an error in perception^4^. However, they represent bias, defined as idiosyncratic interpretations of emotions that are not shared by other perceivers and instead stem from the unique context, namely, perceiver’s perceptual readiness arising from personality traits, values, personal experiences, or social knowledge. As such, noise can be informative of the perceiver and the perceived.

More signal decoding does not necessarily entail less noise perception. Signal decoding (i.e., identifying accurately the target emotion) is theoretically independent from noise perception (i.e., identifying inaccurately secondary emotions)^24^. That an individual perceives some level of sadness in an expression primarily considered angry does not have to impinge on the perception of anger. Yet, the tendency to perceive also sadness in the angry expression is likely linked to individual differences among perceivers^4^. It is thus useful to assess additional perceptions along the main target emotion instead of capturing only one crude form of inaccuracy (i.e., mistaking one emotion for another). In fact, separate assessments of signal decoding and noise perception in emotion recognition tasks are more effective in revealing associations with personality traits than assessments based on simple hit rates^24^.

Both signal and noise perception have implications for social interactions^25^. Whereas signal decoding is associated with positive experiences (i.e., perceiving interaction partners as well-intentioned, reporting less negative affect), noise perception is associated with negative experiences (i.e., dissatisfaction with social interactions, increased negative affect, decreased positive affect)^26,27^. Noise perception, however, is linked not only to higher negative affect but also to higher positive affect, suggesting that participants who perceive more noise may approach social interactions in a more volatile emotional frame and thus feel more intense emotions^22^. Noise perception is also negatively associated with the degree to which participants report feeling understood, accepted, supported, and satisfied with their social interaction, and with the degree to which they view the interactant as open and expressing positive emotions. Finally, although signal decoding has a pervasive impact on intimate relationships, noise perception has a pervasive impact across all types of relationships^22,26^; hence, all types of interactions can be disrupted by noise perception. Thus, it is as important to study the association between narcissism and noise perception as the association between narcissism and signal decoding.

Existing research makes it difficult to formulate unequivocal hypotheses linking narcissism with signal decoding. Generally, agentic narcissists pay less attention to others’ faces and, as a result, struggle to remember and recognize people and their surrounding environment^28^. Given that attention to faces is a necessary prerequisite for accurately recognizing facial emotional expressions, high narcissists’ self-focus and inattention to others might have a negative impact on their emotion recognition, including signal decoding. Further, agentic narcissists are disagreeable^5^ and not particularly trustful^29^, unlike high signal decoders^24^. Even though high (vs. low) agentic narcissists elicit positive first impressions on others and are found attractive and likeable at zero-acquaintance, they do not report particularly positive experiences in intimate relationships, and neither like nor are consistently liked by their peers^30^, which would be the case for high signal decoders.

In contrast, high (vs. low) communal narcissists present themselves as trustful and endorsing positive generalized beliefs about others^29^, as would be expected of high signal decoders^26^. Yet, evidence exploring the actual links between communal narcissism and popularity among peers is scarce and mixed^9^. It is thus not clear that communal narcissism is linked to signal decoding, which fosters smooth interactions and increases mutual liking. Given the inconclusive findings, we adopted an exploratory approach in Study 1 regarding the associations of agentic and communal narcissism with signal decoding.

More research findings allow linking narcissism with noise perception. The latter is associated with various perceiver traits, some of which, such as insecure attachment and greater subjective social status^22,24^, characterize high agentic narcissists. A positive association between noise perception and agentic narcissism might be due to the fact that agentic narcissists are hypervigilant to self-threat^31,32^, which might make them more susceptible to perceiving and inferring secondary emotions in others’ faces (i.e., noise perception), such as scorn or disgust. Such a process would likely disrupt interactions with other people. Indeed, high narcissists experience interpersonal interactions more unfavorably: They think negatively of others^33^, show low empathy^34^, and regard others as colder, distrustful, and threatening^5,35^. They often feel misunderstood or rejected by their interaction partners, perceive the partners as cold, report a greater number of daily interpersonal transgressions perpetrated against them, are easily offended, and often feel victimized and vengeful^36,37^ (see also^38,39^). This maps well onto the characteristics of high noise perceivers: Noise perception is negatively associated with the degree to which participants report feeling understood, accepted, supported, and satisfied with the social interaction, and with the degree to which the interactant is seen as open and expressing positive emotions^22,26^.

Also, specific emotional and interpersonal consequences of noise perception point to the possibility that agentic narcissism is associated with it, as agentic narcissists often show mood variability and reactivity to interpersonal events, that is, intense negative and positive affect^5,40^. These characteristics are typical for high noise perceivers, who experience not only more negative affect but also more positive affect^24,26^. Still, given the scarcity of empirical findings, our approach is exploratory in Study 1.

Communal narcissists are driven by the same motives as agentic narcissists (i.e., grandiosity, entitlement, status, power), but display these motives in the communal domain^6^. Unlike their agentic counterparts, communal narcissists magnify their prosociality (e.g., helpfulness, care, trust, positive generalized beliefs about others)^6,9^, atypically for high noise perceivers. However, their claims are not met with consensus by objective observers^41^, suggesting that communal narcissists might not be particularly prosocial, which is rather typical for high noise perceivers. High communals claim to like others better^9^, also atypically for high noise perceivers, but it is not clear that they are liked back^9^. Hence, it remains unclear whether high communals display characteristics of high versus low noise perceivers. Based on these mixed self- and other-reported characteristics, we opted for an exploratory approach to the association between agentic and communal narcissism and noise perception in Study 1.

We conducted two studies testing German and Polish samples. We measured signal decoding, the correct ratings of the focal emotion that corresponds to the expresser’s state, and noise perception, the ratings of additional emotions that were not intended to be expressed, in emotion recognition among participants differing in levels of agentic and communal narcissism. We adopted an exploratory approach to the associations of narcissism and the emotion recognition indices in Study 1, and a confirmatory approach in Study 2.

## Results

### Study 1

In Study 1, we explored the associations between grandiose (agentic and communal) narcissism, and emotion recognition—both signal decoding and noise perception—in a laboratory in Germany. One day prior to the laboratory session, participants (*n* = 147) completed an online demographics questionnaire and measures of agentic and communal narcissism. On the next day, in the laboratory, participants completed an emotion recognition task where each rated 48 photographs (12 stimuli per emotion) out of a pool of 144 naturalistic photographs from the Assessment of Contextualized Emotions-faces set (ACE-faces)^22,27^. Each photograph depicted a target person at its center expressing one of four emotions: happiness, sadness, anger, disgust. Participants rated the emotional expression of the target person on calmness, fear, anger, surprise, disgust, sadness, and happiness (1 = *not at all*, 7 = *very much*) following stimulus presentation. We defined signal decoding as the rating on the emotion scale that corresponded to the intended emotion shown by the target person (i.e., rating on the sadness scale for the person displaying a sad face). We defined noise perception as the mean of the ratings on the other six emotion scales (i.e., calmness, fear, anger, surprise, disgust, happiness for the target person displaying a sad face). We computed signal decoding and noise perception measures separately for each stimulus.

#### Signal decoding

**Figure 1:**
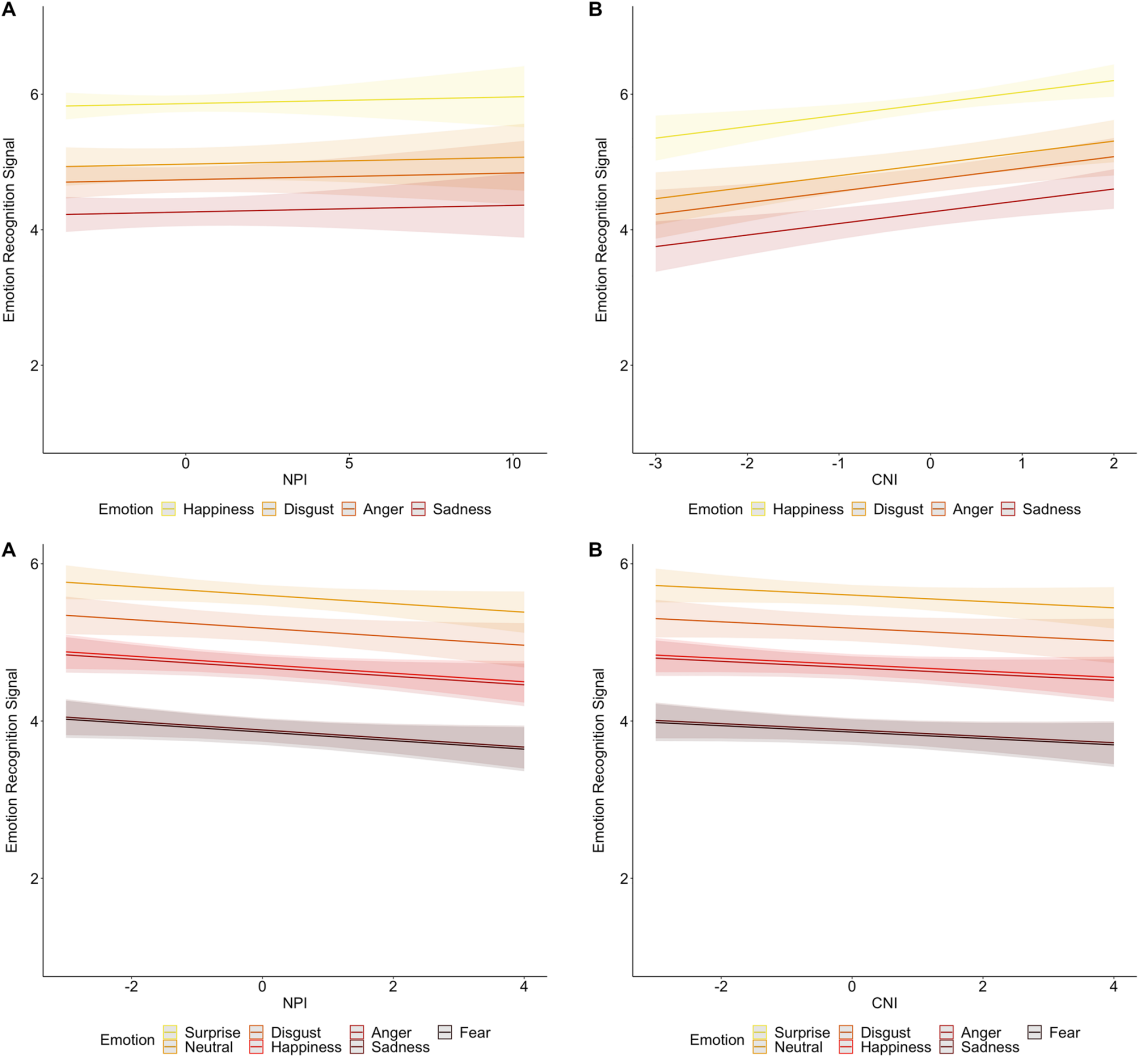
Agentic narcissism (NPI; **A**) and communal narcissism (CNI; **B**) as predictors of signal decoding of each emotional display in Study 1 (top panel) and in Study 2 (bottom panel). Light-colored areas represent 95% error bands.

We tested whether agentic and communal narcissism predicted signal decoding (in two separate analyses) with linear mixed-effect modeling (LMM). Agentic narcissism did not predict signal decoding (see Table 1 top left and Fig. 1 top left—A for effects of narcissism; see Supplementary Table S2 for full results of the model).However, communal narcissism predicted signal decoding: High (vs. low) communal narcissists were better at signal decoding (Table 1 top right and Fig. 1 top right).

**Table 1:** Results of mixed effect models in Study 1 (top panel) and Study 2 (bottom panel). ICC = intraclass correlation coefficient. The table only presents the effects of narcissism. For the full results, including the effects of emotion type, see Supplementary Tables S2 (Study 1) and S4 (Study 2).

Predictors	Signal model agentic narcissism					Signal model communal narcissism				
	Estimates	SE	95% CI	p	df	Estimates	SE	95% CI	p	df
*Study 1*										
(Intercept)	4.96	0.06	4.84–5.08	** < .001**	144.68	4.96	0.06	4.84–5.08	** < .001**	144.96
Agentic Narcissism	0.01	0.02	−0.03–0.05	** < **.646	145.02					
Communal Narcissism						0.17	0.05	0.07–0.27	** < .002**	145.01
ICC	0.30					0.29				
N	147_id_					147_id_				
Observations	7053					7053				
Marginal R^2^/Conditional R^2^	0.115/0.384					0.125/0.382				
*Study 2*										
(Intercept)	4.87	0.03	4.81–4.94	** < .001**	517.23	4.87	0.03	4.79–4.93	** < .001**	517.64
Agentic Narcissism	−0.05	0.03	−0.11–0.001	** < **.063	517.99					
Communal Narcissism						−0.04	0.03	−0.10–0.02	** < **.165	518.43
ICC	0.23					0.23				
N	521_id_					521_id_				
Observations	14,496					14,496				
Marginal R^2^/Conditional R^2^	0.142/0.343					0.141/0.343				

Significant values are in bold.

#### Noise perception

**Figure 2:**
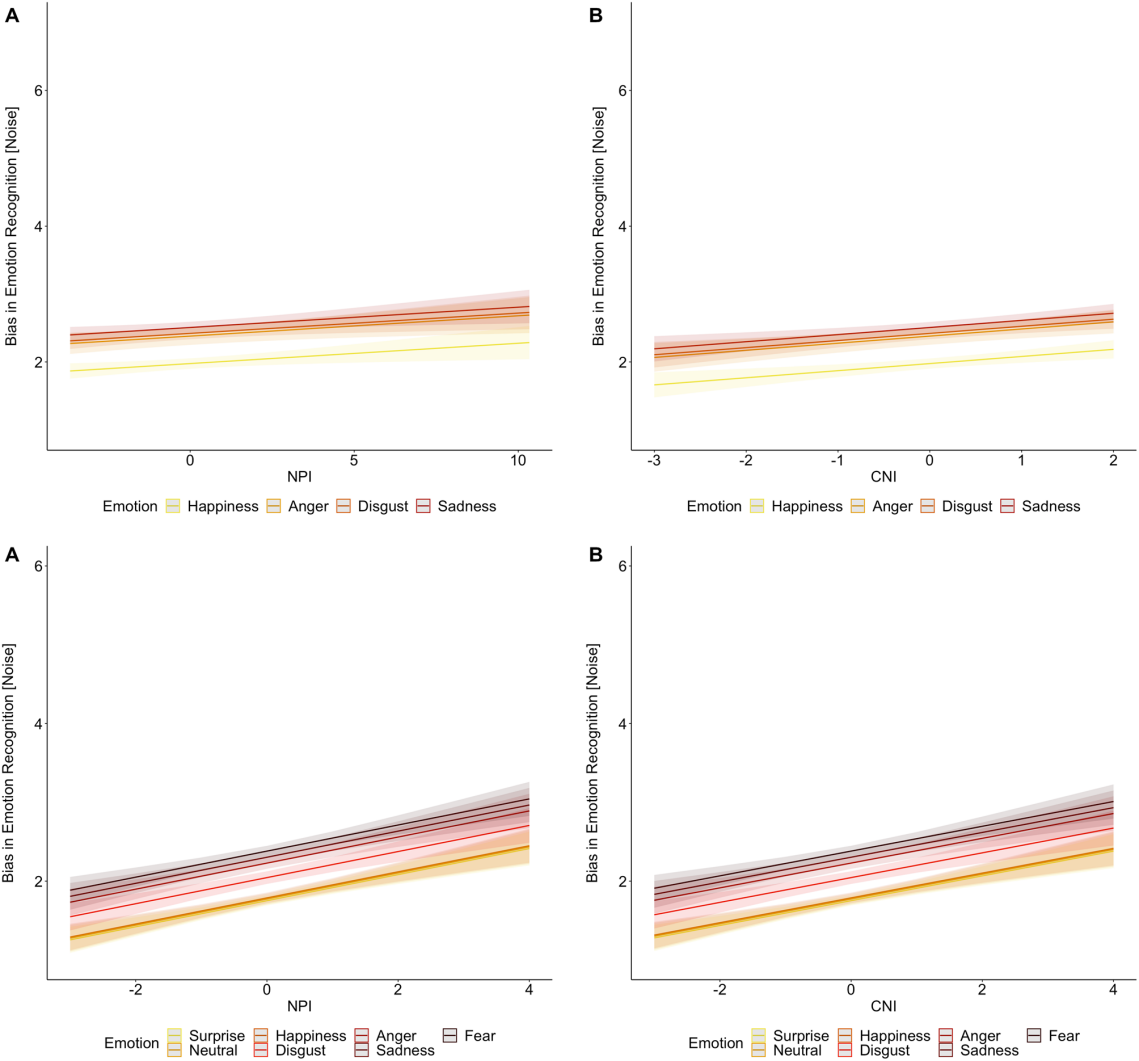
Agentic narcissism (NPI; **A**) and communal narcissism (CNI; **B**) as predictors of noise perception for each emotional display in Study 1 (top panel) and in Study 2 (bottom panel). Light-colored areas represent 95% error bands.

We then tested whether agentic and communal narcissism predicted noise perception, again with LMM. Agentic narcissism predicted noise perception: High (vs. low) agentic narcissists were significantly higher in noise perception (see Table 2 top left and Fig. 2 top left for effects of narcissism; see Supplementary Table S3 for full results of the model). Communal narcissism also predicted noise perception: High (vs. low) communal narcissists perceived more noise (Table 2 top right and Fig. 2 top right).

**Table 2:** Results of mixed effect models in Study 1 (top panel) and Study 2 (bottom panel): Agentic and communal narcissism predict noise perception note. ICC = intraclass correlation coefficient. The table only presents the effects of narcissism. For the full results, including the effects of emotion type, see Supplementary Tables S3 (Study 1) and S5 (Study 2).

Predictors	Noise Model Agentic Narcissism					Noise Model Communal Narcissism				
	Estimates	SE	95% CI	p	df	Estimates	SE	95% CI	p	df
*Study 1*										
(Intercept)	2.32	0.04	2.24–2.40	** < .001**	144.26	2.32	0.04	2.25–2.40	** < .001**	143.89
Agentic Narcissism	0.03	0.01	0.01–0.05	** < .010**	136.88					
Communal Narcissism						0.10	0.03	0.05–0.16	** < .001**	137.10
ICC	0.56					0.55				
N	147_id_					147_id_				
Observations	7053					7053				
Marginal R^2^/Conditional R^2^	0.097/0.602					0.109/0.597				
*Study 2*										
(Intercept)	2.04	0.03	1.98–2.10	** < .001**	464.42	2.04	0.03	1.98–2.10	** < .001**	435.90
Agentic Narcissism	0.17	0.03	0.11–0.22	** < .001**	555.67					
Communal Narcissism						0.16	0.03	0.11–0.21	** < .001**	560.20
ICC	0.67					0.67				
N	521_id_					521_id_				
Observations	14,496					14,496				
Marginal R^2^/Conditional R^2^	0.113/0.708					0.109/0.708

### Study 2

In Study 2, we switched from an exploratory to a confirmatory approach. We hypothesized that communal narcissism would predict signal decoding, and that both agentic and communal narcissism would predict noise perception.

We tested the hypotheses with a larger sample (*n* = 520) and in another culture (i.e., Poland) online. Further, we used different stimulus materials with a wider array of emotional expressions for generalizability purposes. Finally, we balanced our design for gender.

As in Study 1, participants first completed a demographic questionnaire and measures of agentic and communal narcissism. Next, they completed an emotion recognition task preceded by a short training session. This task used 28 photographs of faces looking straight ahead from *The Karolinska Directed Emotional Faces* (KDEF)^42^. Each photograph had been selected to be neither obvious nor too ambiguous to recognize and displayed one of seven emotional facial expressions (happiness, sadness, anger, fear, disgust, neutral, surprise). Participants rated the emotional expression on fear, anger, surprise, disgust, sadness, and happiness (1 = *not at all*, 7 = *very much*) following stimulus presentation.

#### Signal decoding

We tested whether agentic and communal narcissism separately predicted signal decoding, as in Study 1 (i.e., using LMM). Like in Study 1, agentic narcissism did not predict signal decoding (see Table 1 bottom left and Fig. 1 bottom left for effects of narcissism; see Supplementary Table S4 for full results of the model). However, contrary to Study 1, communal narcissism also did not predict signal decoding (Table 1 bottom right and Fig. 1 bottom right].

#### Noise perception

Next, we examined, via LMM, whether agentic and communal narcissism predicted noise perception. As in Study 1, agentic narcissism predicted noise perception: High (vs. low) agentic narcissists were significantly higher on noise perception (see Table 2 bottom left and Fig. 2 bottom left for effects of narcissism; see Supplementary Table S5 for full results of the model). Similarly, replicating the findings of Study 1, communal narcissism predicted noise perception: High (vs. low) communal narcissists were significantly higher on noise perception (Table 2 bottom right and Fig. 2 bottom right).

To summarize, unlike Study 1, neither form of grandiose narcissism predicted signal decoding. Yet, consistent with Study 1, both agentic and communal narcissism predicted noise perception.

## Discussion

We investigated the association between narcissism (agentic and communal) on the one hand and signal decoding as well as noise perception on the other. In two studies, we implemented validated stimuli and relied on culturally distinct samples. We began with an exploratory approach in Study 1, using a stimulus pool that represented expressions of four emotions (happiness, sadness, anger, disgust) and recruiting a sample of moderate size in Germany. We switched to a confirmatory approach in Study 2 to test the replicability of Study 1 findings. Here, our stimuli represented expressions of a wider range of emotions (happiness, sadness, anger, fear, disgust, neutral, surprise), and our sample, recruited in Poland, was larger and gender-balanced.

In Study 1, communal, but not agentic, narcissism positively predicted signal decoding. However, this pattern of results did not replicate in Study 2, where neither agentic nor communal narcissism was associated with signal decoding. The sample of Study 1 was smaller and comprised mostly young women, whereas that of Study 2 was larger and gender-balanced. Hence, on the basis of the Study 2 sample characteristics, we presume a null relation between both forms of grandiose narcissism and signal decoding. Another explanation for why the effect of communal narcissism on signal did not replicate in Study 2 is that the Study 2 stimuli presented individuals alone in contrast to the social context of Study 1 ACE stimuli where one person was surrounded by two others. Perception of stimuli in the absence of social context relates less strongly to personality measures^4^. Altogether, the association between grandiose narcissism and signal decoding warrants further investigation.

Across studies, both narcissism forms positively predicted noise perception. This may explain, at least in part, grandiose narcissists’ impaired quality of social interactions^22^. In particular, signal decoding entails a more precise assessment of the interactant’s emotional state, and thereby a more suitable reaction to the interactant’s emotional expressions and behaviors. However, noise perception ‘muddles’ the emotional inference, as it taints the discernment of the focal emotion from the interactant’s face and renders it attenuated by one or more additional emotions. Hence, a narcissist’s reaction may be only slightly off and may not be reciprocated by a corrective reaction from the interactant, contributing to an uncomfortable or strained social encounter that might have a cumulative negative influence on relationship quality. Hence, the finding linking grandiose narcissism with noise perception may partly explain why narcissism (at least the agentic form) is associated with relational problems—no matter if the relationship is distant or close^43^. The problems likely arise partly due to increased noise perception. High agentic narcissists (as assessed by NPI) monitor their interpersonal worlds with hostility and suspicion in their efforts to detect and diffuse potential threats to their self-aggrandizement^32,44^. Their hypervigilance likely translates to perceptual readiness to see and infer secondary emotions in addition to the dominant one from others’ faces (i.e., noise perception). This could explain why high narcissists might frequently perceive others as rejecting them which, in turn, might lead them to behave in ways that evoke actual rejection^45^. High noise perception might manifest itself as a hostile attribution bias that causes narcissists to infer negative intent in ambiguous situations^37^. Further investigation of this possibility is warranted.

We conducted additional analyses testing interactions between grandiose narcissism (agentic, communal) and perceptions of specific, as well as positive and negative, emotions (Supplementary Information). Although grandiose narcissism was not consistently linked to increased perception of either negative or positive emotions, it was linked to increased perception of all emotions regardless of their valence. In particular, across studies, high (vs. low) grandiose narcissists rated emotions as higher, on average. In Study 2, high (vs. low) narcissists discriminated signal from noise to a significantly lower degree and perceived all emotions as expressed more intensely regardless of signal or noise. Taken together, even though grandiose narcissists perceive signal emotions correctly, they likely experience difficulty in discerning the predominant emotion from other emotions in facial expressions.

Our research provides insights into the association between grandiose narcissism and emotion recognition ability. Grandiose narcissists perceive more emotions in their interaction partners’ faces than the face objectively reveals. This finding was replicated in two cultures and with different stimulus materials. Our studies, though, only provided correlational support for the association between narcissism and noise perception. We assumed that narcissists’ egocentric exceptionalism and social selfishness^5^ hinder their ability to discern their interactant’s emotion from among several projected “noise” emotions, as narcissists may be less likely to exert effort or consider it worth paying attention to subtle and discreet changes in their partner’s facial expressions. Future research would need to manipulate narcissism^46^ and then examine its effects on emotion recognition strategies. Future research might also consider the role of components of grandiose narcissism, such as admiration and rivalry^35^, and additional forms of narcissism, such as vulnerable narcissism^5^, for emotion recognition. Finally, future research might consider tactics, if not interventions to decrease noise perception among narcissists, which may improve narcissists’ interaction quality.

It has long been recognized that narcissists malfunction in interpersonal and relational settings. Our findings provide one explanation for this phenomenon. Narcissists are susceptible to noise perception, without necessarily being better or worse at signal decoding. Perceiving additional emotions (noise) may disrupt social relationships even more than failing to detect the intended emotion.

## Methods

### Study 1

#### Participants

Based on Hess and colleagues^22^, who implemented the same emotion recognition task (and also measured participants’ traits, such as negative affect, prior to the laboratory session), we used the *simr* package^47^ to run a simulation-based linear mixed model (LMM) power analysis with the *lme4* package^48^. That is, assuming a similar effect size for narcissism as for negative affect, we used the coefficients of the fixed and random effects of an analysis like the planned one to calculate the minimum sample size for Study 1 for achieving at least 80% power at an alpha level of 0.05. The analysis pointed to recruiting at least 130 participants (power curve in Supplementary Fig. S1).

Hedging against attrition, we recruited 152 participants via the Humboldt-Universität zu Berlin participant pool for course credit or via advertisements on Facebook, campus recruitment, and flyers (for 25€). We excluded data from one participant due to an early termination decision, and from four participants due to technical problems (i.e., equipment malfunction) or a clerical error. The final sample consisted of 147 participants (106 women, 38 men, 3 non-binary) between the ages of 17 and 51 years (*M* = 28.2, *SD* = 7.12).

#### Procedure

One day prior to the laboratory session, participants completed an online demographic questionnaire and measures of narcissism in a separate random order. Upon arrival at the laboratory, participants were seated in a comfortable chair and completed the emotion recognition task (along with additional measures, unreported here, collected for different research objectives).

#### Measures

We assessed agentic narcissism with the 15-item German version of the NPI (NPI-15^49^; α = 0.69, the reliability, although low, falls within the typical range for this short measure^50^). Participants choose between two statements, one narcissistic (coded as 1; e.g., “I think I am a special person”) and one non-narcissistic (coded as 0; e.g., “I am no better or worse than most people”). We calculated the total score by summing up the answers.

We assessed communal narcissism with the 16-item Communal Narcissism Inventory (CNI^6^; α = 0.91). Sample items are: “I am the most helpful person I know” and “I will be able to solve world poverty”; 1 = *strongly disagree*, 7 = *strongly agree*). As in prior research^6^, the CNI was moderately correlated with the NPI (*r* = 0.27, *p* = 0.001).

We assessed emotion recognition with the ACE-faces^4,22,27^. The task consists of a series of naturalistic photographs, which had been created using the relived emotion task. Specifically, prior to the recording, each expresser was instructed to remember and to recount as vividly as possible a time when they had felt a particular emotion; apex facial expressions were then selected and validated with regard to the signal emotion by 26 independent raters^22^. Each photograph depicts a target person at its center expressing one of four emotions: happiness, sadness, anger, disgust. The stimulus pool has been used in prior research^22,24,27^. It comprises 144 stimuli. In particular, six female and six male targets display four emotions (anger, sadness, disgust, happiness) in three conditions (alone, with two neutral others, with two others having the same expression; 12 × 4 × 3 = 144). Each participant only saw 1/3 of the stimuli. We created 12 orders via a Latin square to ascertain that an equal number of each expression was presented in each condition.

Participants rated the emotional expression of the target person (presented for 6 s) on calmness, fear, anger, surprise, disgust, sadness, and happiness (1 = *not at all*, 7 = *very much*) following stimulus presentation. We defined signal decoding as the rating on the emotion scale that corresponded to the intended emotion shown by the target person (i.e., rating on the sadness scale for the person displaying a sad face). We defined noise perception as the mean of the ratings on the other six emotion scales (i.e., calmness, fear, anger, surprise, disgust, happiness for the target person displaying a sad face). We computed signal decoding and noise perception measures separately for each stimulus.

### Study 2

#### Participants

We used the same strategy for estimating the required sample size as in Study 1. Here, however, we based the estimate of the effects of agentic and communal narcissism on the effect obtained for agentic narcissism on noise perception in Study 1 (which had been the smallest of the significant effects). Aiming for at least 95% power at an alpha level of 0.05, we conducted a simulation-based LMM power analysis with the *simr* package^47^. The suggested minimum sample size was 400 participants (power curve in Supplementary Fig. S2).

To account for potential data loss (of up to 20%) in online studies due to technical problems^51^, we tested 520 participants online (261 women, 259 men). We recruited them via social media posts or snowballing (71%) and via the Polish research recruitment platform ABR SESTA (https://abrsesta.com/pl/; 29%). Participants ranged in age from 15 to 65 years (*M* = 30.01, *SD* = 11.42), and received a payment of 3 PLN ($0.80).

#### Procedure

Following demographic information, assessment of the two forms of narcissism, and measures irrelevant to this article (i.e., mood), participants completed the emotion recognition task preceded by a short training session. We used a training session to ensure that participants understood the instructions, as this was an online study. Usually, no training is required for the emotion recognition task^22^.

#### Measures

We assessed agentic narcissism with the 13-item NPI (NPI-13^52^; Polish version^53^; α = 0.89, e.g., “I like to look at myself in the mirror”; 1 = *strongly disagree*, 7 = *strongly agree*). We assessed communal narcissism with the CNI^6^ (Polish version^54^; α = 0.93; e.g. “I am the most caring person in my social surrounding”; 1 = *strongly disagree*, 7 = *strongly agree*).

We measured emotion recognition with stimuli from *The Karolinska Directed Emotional Faces* (KDEF)^42^. The stimuli had been created using the relived emotion task and later validated by 272 independent raters. Since its creation, KDEF has been widely implemented. (For documentation covering hit rates, means, and dispersion measures of intensity and arousal ratings^55^). First, during the training session, participants saw two photographs, followed by rating scales (see below). Next, in the main session, we presented participants with photographs of faces looking straight ahead. We included 28 photographs (14 female faces, 14 male faces), each displaying one of seven emotional facial expressions (happiness, sadness, anger, fear, disgust, neutral, surprise) from the validated subset of 490 KDEF photographs^55^. To ensure that the selected emotions would be neither obvious nor ambiguous to recognize, we randomly selected the 28 photos from among those that featured 50–80 percent of correct rates in the KDEF validation study^55^. We presented each photograph for 6 s, with participants subsequently rating the degree to which the person in the photograph appeared fearful, angry, disgusted, happy, neutral, sad, and surprised (1 = *not at all*, 7 = *very much*). We defined signal decoding as the rating on the emotion scale that corresponded to the intended emotion shown by the target person (i.e., rating on the sadness scale for the person displaying a sad face). We defined noise perception as the mean of the ratings on the other six emotion scales (i.e., happiness, anger, fear, disgust, neutral, surprise for the target person displaying a sad face). We computed signal decoding and noise perception measures separately for each stimulus.

### Statistical analyses

In both studies, we tested whether agentic and communal narcissism predicted signal decoding (in two separate analyses) with linear mixed-effect modeling (LMM; *lme4* and *lmerTest*)^48,56^. Each time the design included two factors. The first factor was continuous and involved agentic and communal narcissism (with the NPI and CNI, correspondingly, centered on the sample’s means). The second, a within-person factor, was emotion type involving repeated contrast coding, so that slopes indicated differences between consecutive factor levels (e.g., in Study 1 model of noise: anger—happiness, disgust—anger, sadness—disgust). We included random intercepts for participants and specified maximally random slopes^57^. We report results pertaining to the first factor in the main body of the manuscript and in Tables 1 and 2. We report the results of the full models, including differences in participants’ recognition—signal decoding and noise perception—of various emotions in the General Emotion Recognition section of Supplementary Information and Supplementary Tables S2–S5. For analyses exploring differences between narcissists’ recognition of specific emotions and generally positive/negative emotions, the effects of target gender, and other factors, see Other Exploratory Analyses in Supplementary Information. Similarly, for additional analyses testing whether high (vs. low) grandiose narcissists rated all emotions higher on average, and whether high (vs. low) grandiose narcissists rated signal emotions as higher than noise emotions, see Other Exploratory Analyses in Supplementary Information.

### Ethical approval

The studies were conducted in accordance with human ethics guidelines and approved by the respective institutions: Study 1 by the Institutional Review Board committee at Humboldt-Universität zu Berlin, Germany (#2019-11) and Study 2 by the Ethics Committee at the Institute of Applied Psychology at Jagiellonian University, Poland (#122/2022). All study procedures involving human participants were in accordance with the ethical standards of the institutional research committee and with the 1964 Helsinki declaration and its later amendments or comparable ethical standards. All participants provided informed consent prior to participation in the studies. We did not preregister the studies.

### Supplementary Information

Supplementary Information.

## Data Availability

We share stimulus materials, and codes at https://osf.io/srmwh/?view_only=49a660961381400d86dd8d06811c2b94. Data are available at https://osf.io/fd6ra?view_only=49a660961381400d86dd8d06811c2b94 (Study 1) and https://osf.io/4g67m?view_only=49a660961381400d86dd8d06811c2b94 (Study 2).
