# Supplementary material for: Narcissism predicts noise perception but not signal decoding in emotion
Source: Sci Rep. 2023 Sep 2;13:14457. doi: 10.1038/s41598-023-41792-0 (PMC10475012; doi:10.1038/s41598-023-41792-0)
Supplement: Supplementary file 1 — Supplementary Information. [file 41598_2023_41792_MOESM1_ESM.docx]

**SUPPLEMENTARY INFORMATION**

**Narcissism Predicts Noise Perception but not Signal Decoding in Emotion**

**Lists of All Variables Collected in Each Study (in Order of Administration)**

***Study 1***

Gender, age, education, relationship status, the longest relationship, use of smartphone and social media, Narcissistic Admiration and Rivalry Questionnaire short version (NARQ)^[1]^, Hypersensitive Narcissism Scale (HSNS)^[2]^, Communal Narcissism Inventory (CNI)^[3]^, German version on the Single-Item Self-Esteem Scale (G-SISE^[4]^; SISE^[5]^), German short scale to measure the need for cognitive closure (NCC)^[6],[7]^, Big Five Inventory-SOEP (BFI-S)^[8]^, Toronto alexithymia scale (TAS-20)^[9]^, Brief Pathological Narcissism Inventory – vulnerability^[10]^, Narcissistic Vulnerability Scale (NVS)^[11]^, Narcissistic Grandiosity Scale (NGS)^[12]^, Social Media Disorder Scale (SMD) ^[13]^, Narcissistic Personality Inventory (NPI) ^[14]^, Emotion Recognition Task: Assessment of Contextualized Emotions-faces (ACE-faces) ^[15]^, Heart rate, Skin conductance and Facial electromyography (EMG) to assess emotional mimicry, Emotional contagion, Emotional empathy – one question (MET) ^[16]^, Interpersonal closeness^[17]^, Vulnerability Scale-state narcissism (NVS) ^[11]^, Narcissistic Grandiosity Scale-state narcissism (NGS) ^[12]^, Analytic-Holistic thinking (AHS) ^[18]^, Diary questions^[19]^ (additional: vulnerable narcissism – one item from PNI-S, adapted for diary^[10]^, state narcissistic entitlement – one item from SD3, adapted for diary^[20]^).

***Study 2***

Gender, age, native language, first four items from General Mood Scales (GMS) ^[21]^, Hypersensitive Narcissism Scale (HSNS^[2]^; Polish version^[22]^), Narcissistic Personality Inventory (NPI-13^[23]^; Polish version^[24]^), Communal Narcissism Inventory (CNI-16^[3]^; Polish version^[25]^), Emotion Recognition Task: The Karolinska Directed Emotional Faces (KDEF) ^[26]^, fifth, sixth, seventh and eighth item from General Mood Scales (GMS) ^[21]^.

As listed above, Study 1 included exploratorily a variety of narcissism measures, such as a clinical scale (Brief Pathological Narcissism Inventory^[10]^) and infrequently used scales (NVS^[11]^, NGS^12]^). In Study 2, we narrowed down the scope of measures and tested the replicability of our findings with commonly used scales that evinced significant associations with emotion recognition in Study 1.

**Sample Size estimation**

*
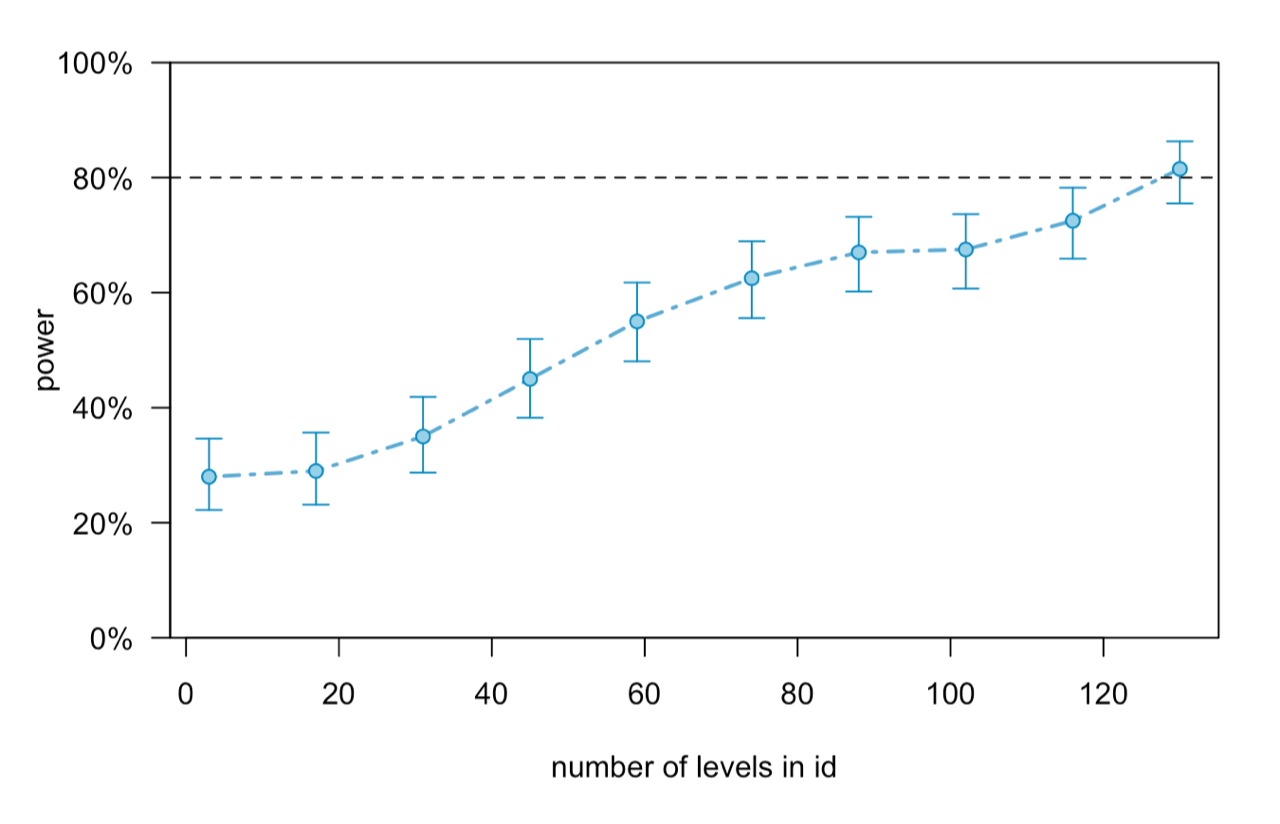
*We ran simulation-based linear mixed model (LMM) power analyses with the *lme4* package^[27]^ to estimate required sample sizes in Study 1 and Study 2. We present resulting power curves in Figure S1 and Figure S2, respectively. *
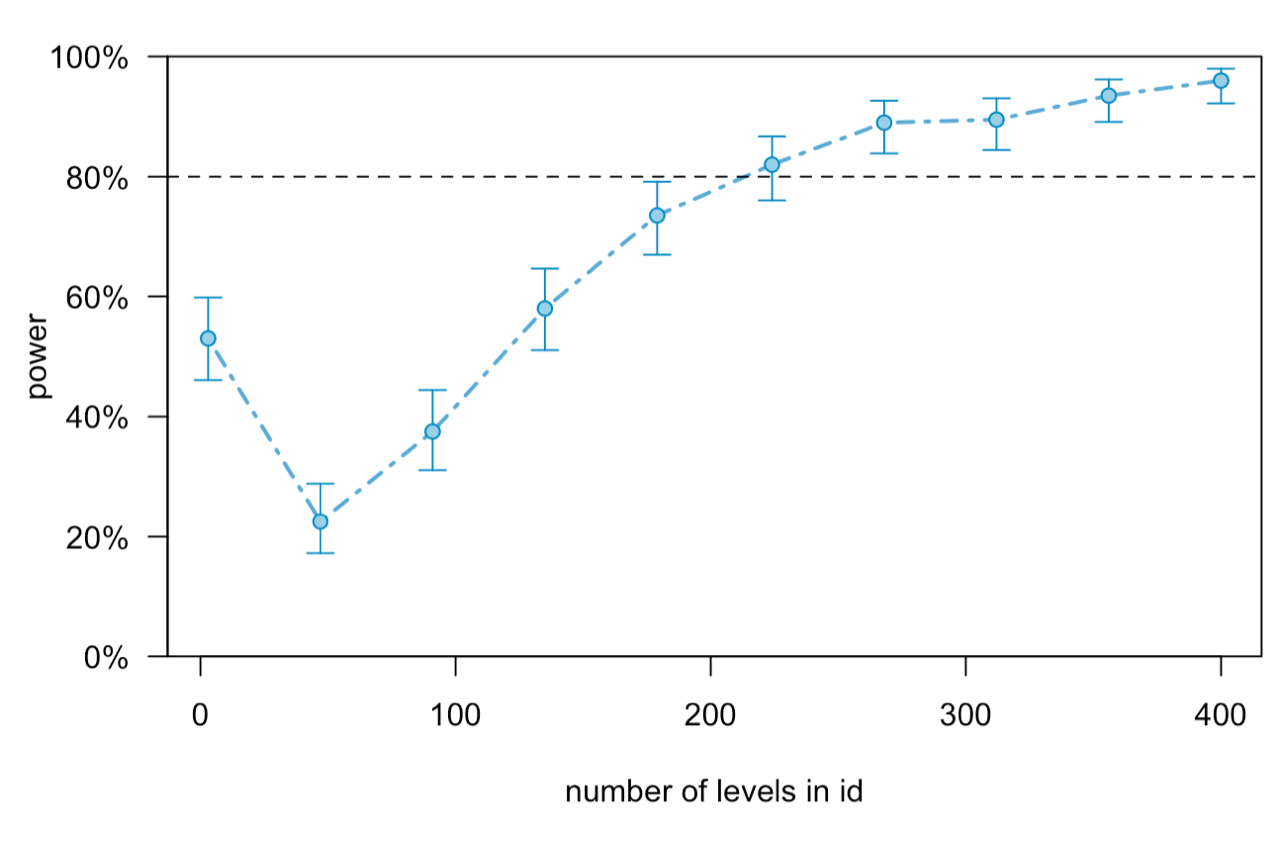
***Figure S1.** Power curve for Study 1

**Figure S2.** Power curve for Study 2.

**General Emotion Recognition**

There is no consensus on which emotions are easier to recognize from facial expressions^[28], [29]^. Surprise, disgust, and happiness have emerged as easier to recognize in some studies, whereas sadness and fear have emerged as easier to recognize in other studies^[29], [30], [31], [32]^.

**Study 1**

***Signal Decoding***

The emotion type effects were significant (Table S2). Participants were lower on signal decoding when the target person displayed disgust than happiness, anger than disgust or happiness, and sadness than anger, disgust, or happiness.

***Noise Perception***

The emotion type effects were significant (Table S3). Participants were higher on noise perception when the target person displayed anger than happiness, and sadness than happiness, anger, or disgust.

***Summary***

Participants were better in recognizing happiness than negative emotions. This finding is consistent across emotion recognition indices (signal decoding, noise perception). Additionally, participants were worse at perceiving sadness, as indicated by least signal decoding and most noise perception for sad expressions. Lastly, participants recognized anger and disgust intermediately well.

**Study 2**

***Signal Decoding***

Participants manifested less signal decoding when the target person displayed (1) a neutral face than surprise, (2) disgust than a neutral face, or surprise, (3) happiness than disgust, a neutral face, or surprise, and (4) sadness or fear than anger, happiness, disgust, a neutral face, or surprise (Table S3).

***Noise Perception***

Participants exhibited more noise perception when the target person (1) displayed disgust than happiness, neutral, or surprise, (2) anger than disgust, happiness, neutral, or surprise, (3) sadness than anger, disgust, happiness, neutral, or surprise, and (4) fear than sadness, anger, disgust, happiness, neutral, or surprise (Table S5).

***Summary***

Unlike Study 1, happiness was not the emotion that participants recognized best. Instead, participants were better in recognizing surprise (not tested in Study 1), as indicated by least noise perception and most signal decoding, than the remaining emotions. Happiness followed surprise in that it was recognized with similarly little noise perception, though with significantly less signal than surprise. Consistent with Study 1, participants were worst at perceiving sadness, as indicated by least signal decoding and most noise perception for sad expressions.

**Other Exploratory Analyses**

**Study 1**

***Congruency Conditions***

The target person was depicted either alone (individual condition) or accompanied by a group of two male or two female individuals. The two accompanying individuals expressed either the same emotion as the target person (congruent condition) or a neutral face (incongruent condition). Using a Latin Square design, we created 12 parallel orders of 48 stimuli for each emotion: six congruent, six incongruent, and six individual male stimuli, as well as six congruent, six incongruent, and six individual female stimuli. The congruency condition did not significantly affect signal detection or noise perception, and controlling for it did not alter the results. Thus, we do not report these analyses.

***Differences between Narcissists’ Recognition of Specific Emotions***

We also explored differences in narcissists’ recognition (both signal decoding and noise perception) between different emotions. We found no significant interaction between emotion and agentic or communal narcissism, and thus we do not report pertinent results. The results did not change when we exchanged emotion with emotion category (with only two levels: positive and negative). It was not the case that people scoring high in narcissism were better in recognizing negative compared to positive emotions nor did they see more negative emotions in positive facial expressions than positive emotions in negative facial expressions.

***General Bias of Narcissists’ Perception of Emotions***

We repeated our main mixed-effects models with an average emotion rating as a dependent variable to test whether high (vs. low) grandiose (communal and agentic) narcissists rated all emotions higher on average. Indeed, high (vs. low) grandiose narcissists saw more emotions independent of the target’s emotional expression (agentic narcissism: *b* = .02, *SE* = 0.01, *t* = 2.23, *p* = .028; communal narcissism: *b* = .10, *SE* = 0.03, *t* = 3.86, *p* < .001). Next, we repeated our main mixed-effects models with an average signal rating minus average noise rating as a dependent variable to test whether high (vs. low) grandiose narcissists rated signal emotions as higher (or lower) than noise emotions. Narcissism of either form did not predict this outcome (i.e., the difference between perceived intensity of signal and noise) and thus we did not report pertinent results. Altogether, even though, high grandiose narcissists perceive signal emotions correctly (or at least not less correctly than low grandiose narcissists), they likely experience difficulties in discerning the predominant emotion from other emotions in facial expressions.

**Study 2**

***Target Gender***

We repeated these analyses adding target gender as an additional factor. The effects of agentic narcissism and communal narcissism on signal decoding and noise perception remained unchanged.

***Differences between Narcissists’ Recognition of Specific Emotions***

As in Study 1, in additional analyses, we explored differences in narcissists’ recognition (both signal decoding and noise perception) between emotions and found no significant interaction between emotion and agentic or communal narcissism, and thus we do not report pertinent results. Again, similar to Study 1, results did not change when we exchanged emotion with emotion category (with only two levels: positive and negative). It was not the case that participants scoring high on narcissism were better in recognizing negative compared to positive emotions (except for communal narcissists who were slightly better in recognizing positive compared to negative emotions, *b* = .08, *SE* = 0.04, *t* = 1.97, *p* = .049—an effect absent from Study 1) nor did they see more negative emotions in positive facial expressions than positive emotions in negative facial expressions.

***General Bias of Narcissists’ Perception of Emotions***

Again, we found that high (vs. low) grandiose (communal and agentic) narcissists saw more emotions independent of the target’s emotional expression (communal narcissism: *b* = .13, *SE* = 0.02, *t* = 6.01, *p* < .001, and agentic narcissism: *b* = .13, *SE* = 0.02, *t* = 6.16, *p* < .001). We also found that narcissism (of both forms) significantly negatively predicted the difference between perceived intensity of signal and noise (communal narcissism: *b* = -.17, *SE* = 0.04, *t* = - 4.39, *p* < .001, and agentic narcissism: *b* = -.18, *SE* = 0.04, *t* = -4.87, *p* < .001). These findings indicate that high (vs. low) narcissists discriminated signal from noise to a significantly lower degree, perceiving all emotions as expressed more intensely regardless of signal or noise. As mentioned previously,, even though, grandiose narcissists perceive signal (at least as correctly as low grandiose narcissists), they probably experience difficulties in discerning the predominant emotion from other emotions in facial expressions.

**General Discussion**

The results of the two studies produced inconsistent results regarding emotions easiest to recognize. In Study 1, participants were better in recognizing happiness than negative emotions, which corroborates earlier reports^[29], [33]^. This finding was consistent across the two components of emotion recognition—signal decoding and noise perception—in Study 1, but was only partially replicated in Study 2. In that study, participants recognized happiness with similarly little noise perception, yet significantly less signal decoding, than surprise. Also in Study 2, participants were better in recognizing surprise (which was absent from Study 1) than the remaining emotions, as indicated by least noise perception and most signal decoding. This result accords with some earlier findings^[32]^. Across both studies, participants were worst at recognizing sadness, as indicated by least signal decoding and most noise perception for sad expressions, a finding consistent with the literature^[29], [30], [31], [32]^.

**References**

1. Back, M. D. et al. Narcissistic admiration and rivalry: Disentangling the bright and dark sides of narcissism. J. Pers. Soc. Psychol. **105**, 1013–1037 (2013).
2. Hendin, H. M. & Cheek, J. M. Assessing hypersensitive narcissism: a reexamination of Murray’s Narcissism Scale. *J. Res. Pers.* **31**, 588–599 (1997).
3. Gebauer, J. E., Sedikides, C., Verplanken, B. & Maio, G. R. Communal narcissism. *J. Pers. Soc. Psychol.* **103**, 854–878 (2012).
4. Brailovskaia, J. & Margraf, J. How to measure self-esteem with one item? Validation of the German single-item self-esteem scale (G-SISE). *Curr. Psychol*. **39**, 2192–2202 (2020).
5. Robins, R. W., Hendin, H. M. & Trzesniewski, K. H. Measuring global self-esteem: Construct validation of a single-item measure and the Rosenberg Self-Esteem Scale. *Pers. Soc. Psychol. Bull*. **27**, 151–161 (2001).
6. Schlink, S. & Walther, E. Kurz und gut: Eine deutsche Kurzskala zur Erfassung des Bedürfnisses nach kognitiver Geschlossenheit. *Zeitschrift für Sozialpsychologie*, **38**, 153–161 (2007).
7. Webster, D. M. & Kruglanski, A. W. Individual differences in need for cognitive closure. *J. Pers. Soc. Psychol.* **67**, 1049–1062 (1994).
8. Schupp, J. & Gerlitz, J.-Y. Big Five Inventory-SOEP (BFI-S). Zusammenstellung sozialwissenschaftlicher Items und Skalen (ZIS - GESIS Leibniz Institute for the Social Sciences, 2014). https://doi.org/10.6102/zis54
9. Bagby, R. M., Parker, J. D. & Taylor, G. J. The twenty-item Toronto Alexithymia Scale—I. Item selection and cross-validation of the factor structure. *J. Psychosom. Res*. **38**, 23–32 (1994).
10. Schoenleber, M., Roche, M. J., Wetzel, E., Pincus, A. L. & Roberts, B. W. Development of a brief version of the Pathological Narcissism Inventory. *Psychol. Assess*. **27**, 1520–1526 (2015).
11. Crowe, M. L. et al. Development and validation of the Narcissistic Vulnerability Scale: An adjective rating scale. *Psychol. Assess.* **30**, 978–983 (2018).
12. Rosenthal, S. A., Hooley, J. M., Montoya, R. M., van der Linden, S. L. & Steshenko, Y. The Narcissistic Grandiosity Scale: A measure to distinguish narcissistic grandiosity from high self-esteem. *Assessment.* **27**, 487–507 (2020).
13. Van den Eijnden, R. J., Lemmens, J. S. & Valkenburg, P. M. The social media disorder scale. *Comput. Hum. Behav*. **61**, 478–487 (2016).
14. Schütz, A., Marcus, B. & Sellin, I. Die Messung von Narzissmus als Personlichkeitskonstrukt: Psychometrische Eigenschaften einer Lang-und einer Kurzform des Deutschen NPI (Narcissistic Personality Inventory). Diagnostica. 50, 202–218 (2004).
15. Hess, U., Kafetsios, K., Mauersberger, H., Blaison, C. & Kessler, C. L. Signal and noise in the perception of facial emotion expressions: From labs to life. *Pers. Soc. Psychol. Bull*. **42**, 1092–1110 (2016).
16. Dziobek, I. et al. Dissociation of cognitive and emotional empathy in adults with Asperger syndrome using the Multifaceted Empathy Test (MET). *J. Autism Dev. Disord*. **38**, 464–473 (2008).
17. Aron, A., Aron, E. N. & Smollan, D. Inclusion of Other in the Self Scale and the structure of interpersonal closeness. J. Pers. Soc. Psychol. **63**, 596–612 (1992).
18. Choi, I., Koo, M. & Choi, J. A. Individual differences in analytic versus holistic thinking. *Pers. Soc. Psychol. Bull*. **33**, 691–705 (2007).
19. Mauersberger, H., Blaison, C., Kafetsios, K., Kessler, C. L. & Hess, U. Individual differences in emotional mimicry: Underlying traits and social consequences. *Eur. J. Pers.* **29**, 512–529 (2015).
20. Jones, D. N. & Paulhus, D. L. Introducing the short dark triad (SD3) a brief measure of dark personality traits. *Assessment.* **21**, 28–41 (2014).
21. Wojciszke, B. & Baryła, W. Skale do pomiaru nastroju i sześciu emocji. *Czasopismo Psychologiczne,* **11**, 31–47 (2005).
22. Czarna, A. Z., Dufner, M. & Clifton, A.D. The effects of vulnerable and grandiose narcissism on liking-based and disliking-based centrality in social networks. *Journal of Research in Personality,* **50***,* 42–45; 10.1016/j.jrp.2014.02.004 (2014).
23. Gentile, B. et al. A test of two brief measures of grandiose narcissism: The Narcissistic Personality Inventory–13 and the Narcissistic Personality Inventory-16. *Psychol. Assess*. **25**, 1120–1136 (2013).
24. Żemojtel‐Piotrowska, M. A., Piotrowski, J. P. & Maltby, J. Agentic and communal narcissism and satisfaction with life: The mediating role of psychological entitlement and self‐esteem. *Int. J. Psychol*. **52**, 420–424 (2017).
25. Żemojtel-Piotrowska, M., Czarna, A. Z., Piotrowski, J., Baran, T. & Maltby, J. Structural validity of the Communal Narcissism Inventory (CNI): The bifactor model. *Pers. Individ. Differ.* **90**, 315–320 (2016).
26. Lundqvist, D., Flykt, A. & Öhman, A. *The Karolinska Directed Emotional Faces (KDEF)* (Department of Neurosciences Karolinska Hospital, 1998).
27. Bates, D., Maechler, M., Bolker, B. & Walker, S.. Fitting linear mixed-effects models using lme4. *J.Stat Softw.* **67**, 1–48; [10.18637/jss.v067.i01](https://doi.org/10.18637/jss.v067.i01) (2014).
28. Guarnera, M., Hichy, Z., Cascio, M. I. & Carrubba, S. Facial expressions and ability to recognize emotions from eyes or mouth in children. *Eur. J. Psychol*. **11**, 183–196; [10.5964/ejop.v11i2.890](https://doi.org/10.5964/ejop.v11i2.890) (2015).
29. Montagne, B., Kessels, R. P., De Haan, E. H. & Perrett, D. I. The emotion recognition task: A paradigm to measure the perception of facial emotional expressions at different intensities. *Percept. Mot. Skills.* **104**, 589–598 (2007).
30. Ekman, P. & Friesen, W. V. Constants across cultures in the face and emotion. *J. Pers. Soc. Psychol.* **17**, 124–129 (1971).
31. Kohler, C. G. et al. Differences in facial expressions of four universal emotions. *Psychiatry Res.* **128**, 235–244 (2004).
32. Kuusikko, S. et al. Emotion recognition in children and adolescents with autism spectrum disorders. *J. Autism Dev. Disord*. **39**, 938–945 (2009).
33. Castelli, F. Understanding emotions from standardized facial expressions in autism and normal development. *Autism*, *9*(4), 428-449 (2005).
34. Ames, D. R. & Kammrath, L. K. Mind-reading and metacognition: Narcissism, not actual competence, predicts self-estimated ability. *J. Nonverbal Behav.* **28**, 187–209 (2004).
35. Fan, Y. et al. The narcissistic self and its psychological and neural correlates: an exploratory fMRI study. *Psychol. Med.* **41**, 1641–1650 (2011).
36. Wai, M. & Tiliopoulos, N. The affective and cognitive empathic nature of the dark triad of personality. *Pers. Individ. Differ*. **52**, 794–799 (2012).
37. Vonk, J., Zeigler-Hill, V., Mayhew, P. & Mercer, S. Mirror, mirror on the wall, which form of narcissist knows self and others best of all? *Pers. Individ. Differ.* **54**, 396–401 (2013).
38. Konrath, S., Corneille, O., Bushman, B. J. & Luminet, O. The relationship between narcissistic exploitativeness, dispositional empathy, and emotion recognition abilities. *J. Nonverbal Behav.* **38**, 129–143 (2014).
39. Tardif, J., Fiset, D. & Blais, C. Narcissistic personality differences in facial emotional expression categorization. *J. Vis*. **14**, 1444–1444; 10.1167/14.10.1444 (2014).
40. Zhang, H., Wang, Z., You, X., Lü, W. & Luo, Y. Associations between narcissism and emotion regulation difficulties: Respiratory sinus arrhythmia reactivity as a moderator. *Biol. Psychol.* **110**, 1–11 (2015).
41. Vonk, J., Zeigler-Hill, V., Ewing, D., Mercer, S. & Noser, A. E. Mindreading in the dark: Dark personality features and theory of mind. *Pers. Individ. Differ*. **87**, 50–54 (2015).
42. Jauk, E., Freudenthaler, H. H. & Neubauer, A. C. The dark triad and trait versus ability emotional intelligence. *J. Individ. Differ*. **37**, 112–118 (2016).
43. Lobbestael, J., de Bruin, A., Kok, E. & Voncken, M. Through rose coloured glasses: An empirical test of narcissistic overestimation. *Personal. Ment. Health.* **10**, 305–316 (2016).
44. Fossati, A., Somma, A., Pincus, A., Borroni, S. & Dowgwillo, E. A. Differentiating community dwellers at risk for pathological narcissism from community dwellers at risk for psychopathy using measures of emotion recognition and subjective emotional activation. *J. Pers. Disord.* **31**, 325–345 (2017).
45. Turner, N. H. An investigation of emotion recognition ability and metacognitive judgements of emotion recognition performance in trait narcissism. (University of Tasmania, 2017).
46. Amiri, S. & Behnezhad, S. Emotion recognition and moral utilitarianism in the dark triad of personality. *Neuropsychiatr. Neuropsychol.* **12**, 135–142 (2017).
47. Ridderinkhof, A., de Bruin, E. I., Brummelman, E. & Bögels, S. M. Does mindfulness meditation increase empathy? An experiment. *Self Identity.* **16**, 251–269 (2017).
48. Pajevic, M., Vukosavljevic-Gvozden, T., Stevanovic, N. & Neumann, C. S. The relationship between the dark tetrad and a two-dimensional view of empathy. *Pers. Individ. Differ*. **123**, 125–130 (2018).
49. Schimmenti, A. et al. Exploring the dark side of personality: Emotional awareness, empathy, and the Dark Triad traits in an Italian Sample. *Curr. Psychol.* **38**, 100–109 (2019).
50. Mota, S. et al. A comprehensive examination of narcissists’ self-perceived and actual socioemotional cognition ability. *Collabra Psychol.* **5**, 6 (2019).
51. Puthillam, A., Karandikar, S. & Kapoor, H. I see how you feel: How the dark triad recognizes emotions. *Curr. Psychol.* **40**, 3966–3973 (2021).
52. Kajonius, P. J. & Björkman, T. Individuals with dark traits have the ability but not the disposition to empathize. *Pers. Individ. Differ.* **155**, 109716 (2020).
53. Schmitt, H. S. et al. The dark side of emotion recognition–Evidence from cross-cultural research in Germany and China. *Front. Psychol.* **11**, 1132 (2020).
54. Raley, J., & Foster, J. Narcissism and Emotion Recognition. Poster presented at the Association for Research in Personality, 7th Biennial Conference. (July, 2021).
55. Ames, D. R., Rose, P. & Anderson, C. P. The NPI-16 as a short measure of narcissism. *J. Res. Pers.* **40**, 440–450 (2006).
56. Costanzo, M. & Archer, D. Interperting the expressive behavior of others: The Interpersonal Perception Task. *J. Nonverbal Behav.* **13**, 225–245 (1989).
57. Archer, D. P. & Costanzo, M. P. The Interpersonal perception task (IPT–15) (1993).
58. Denecke, F. W. & Hilgenstock, B. *The narcissism inventory*. (Hans Huber, 1989).
59. Bowers, D., Blonder, L. X. & Heilman, K. M. Florida affect battery. (Center for Neuropsychological Studies, Department of Neurology, 1998).
60. Bradley, M. M. & Lang, P. J. Measuring emotion: the self-assessment manikin and the semantic differential. *J. Behav. Ther. Exp. Psychiatry.* **25**, 49–59 (1994).
61. Pincus, A. L. et al. Initial construction and validation of the Pathological Narcissism Inventory. *Psychol. Assess.* **21**, 365–379 (2009).
62. Baron‐Cohen, S., Wheelwright, S., Hill, J., Raste, Y. & Plumb, I. The “Reading the Mind in the Eyes” test revised version: A study with normal adults, and adults with Asperger syndrome or high‐functioning autism. *J. Child Psychol. Psychiatry.* **42**, 241–251 (2001).
63. Tracy, J. L., Robins, R. W. & Schriber, R. A. Development of a FACS-verified set of basic and self-conscious emotion expressions. *Emotion.* **9**, 554 (2009).
64. Gosselin, F. & Schyns, P. G. Bubbles: a technique to reveal the use of information in recognition tasks. *Vision. Res.* **41**, 2261–2271 (2001).
65. Mayer, J. D., Salovey, P., Caruso, D. R. & Sitarenios, G. Measuring emotional intelligence with the MSCEIT V2.0. *Emotion.* **3**, 97–105 (2003).
66. Jonason, P. K. & Webster, G. D. The dirty dozen: A concise measure of the dark triad. *Psychol. Assess.* **22**, 420–432 (2010).
67. Montagne, B., Kessels, R. P., De Haan, E. H., & Perrett, D. I. The emotion recognition task: A paradigm to measure the perception of facial emotional expressions at different intensities. *Percept. Mot. Skills.* **104**, 589–598 (2007).
68. Dziobek, I. et al. Introducing MASC: a movie for the assessment of social cognition. *J. Autism Dev. Disord*. **36**, 623–636 (2006).
69. Scherer, K. R. & Scherer, U. Assessing the ability to recognize facial and vocal expressions of emotion: Construction and validation of the Emotion Recognition Index. *J. Nonverbal Behav.* **35**, 305–326 (2011).
70. Ekman, P. & Friesen, W. V. Constants across cultures in the face and emotion. *J. Pers. Soc. Psychol.* **17**, 124 (1971).
71. Schlegel, K. & Scherer, K. R. Introducing a short version of the Geneva Emotion Recognition Test (GERT-S): Psychometric properties and construct validation. *Behav. Res. Methods.* **48,** 1383–1392 (2016).
72. Miller, J. D. et al. The Five-Factor Narcissism Inventory (FFNI): a test of the convergent, discriminant, and incremental validity of FFNI scores in clinical and community samples. *Psychol. Assess.* **25**, 748 (2013).
73. Schlegel, K., Grandjean, D., & Scherer, K. R. Introducing the Geneva emotion recognition test: an example of Rasch-based test development. *Psychol. Assess.* **26,** 666 (2014).

| **Authors** | **Sample** | **Focus** | **Assessment of Narcissism** | **Assessment of Emotion Recognition** | **Findings on Emotion Recognition** |
| --- | --- | --- | --- | --- | --- |
| Ames & Kammrath (2004)^[34]^ | S1: 143 students (76% male); S2: 164 students (74% male) | trait narcissism | NPI-16 | S1: IPT-15; S2: negotiation task | NP traits had no link to performance but to overestimation of one’s own skill. |
| Fan et al. (2011)^[35]^ | 34 community members (38% male) | trait narcissism | NI | FAB | Compared high versus low NP traits; no differences for emotion recognition task. |
| Wai & Tiliopoulos (2012)^[36]^ | 139 students (24% male) | trait narcissism | NPI | facial emotion recognition involving SAM | NP traits related positively to accuracy in the identification of angry faces. Also, overestimation of one's own abilities. |
| Vonk et al. (2013)^[37]^ | 368 students (24% male) | trait narcissism | NPI, PNI | RMET | NPI (but not PNI) was a negative predictor of RMET scores. |
| Konrath et al. (2014)^[38]^ | S1: 96 students (23% male); S2: 88 MTurkers (41% male) | trait narcissism | NPI-16 (S1), NPI (S2) | RMET and emotion recognition task with stimuli from the UCDSEE | NPI-16 exploitativeness was associated with increased emotion recognition, specifically with recognizing negative emotions |
| Tardif et al. (2014)^[39]^ | 20 healthy subjects (gender not stated) | trait narcissism | NPI | facial expression categorization using Bubbles^[64]^ and a separate expression categorization task involving fully-visible faces | High NP traits were related to slower emotion recognition and looking at different (than low NP traits) parts of the face to recognize fear. |
| Zhang et al. (2015)^[40]^ | 396 adolescents (43% male) | trait narcissism | NPI | MSCEIT | NP traits associated with impaired ability EI but higher self-reported (trait) EI. |
| Vonk et al. (2015)^[41]^ | 929 students (18% male) | trait narcissism | PNI | RMET | PNI (neither Grandiosity nor Vulnerability) had no relation with RMET scores. |
| Jauk et al. (2016)^[42]^ | 540 students (26% male) | trait narcissism | DTDD | MSCEIT - emotion management and emotional relationships (but not Faces) as indicators | NP traits were negatively related to ability EI but not self-reported trait EI in males. |
| Lobbestael et al. (2016)^[43]^ | 85 students (47% male) | trait narcissism | NPI | RMET | No correlation found with RMET scores but with overestimation of own abilities. |
| Fossati et al. (2017)^[44]^ | 126 community members (43% male) | trait (pathological) narcissism | PNI | RMET | Individuals with high (pathological) NP traits had lower scores on RMET, RMET positive items, and RMET negative items by not on neutral items. |
| Turner (2017) ^[45]^ | 60 students (30% male) | trait narcissism | SD3 | ERT | Individuals with high NP traits trendingly worse at recognizing fear, sadness and surprise. Also trendingly worse at recognizing lower intensity emotions. |
| Amiri & Behnezhad (2017)^[46]^ | 120 students (0% male) | trait narcissism | SD3 | stimuli from International Affective Picture System (IAPS) | Individuals with high NP traits are worse at recognizing hatred (but not anger). |
| Ridderinkhof et al. (2017)^[47]^ | 150 community members or students (39% male) | trait narcissism | NPI-16 | RMET | Positive correlation found with RMET scores. |
| Pajevic et al. (2018)^[48]^ | 576 community members (43% male) | trait narcissism | NPI | RMET | No correlation found with RMET scores but with overestimation of own abilities. |
| Schimmenti et al. (2019)^[49]^ | 799 community members (45% male) | trait narcissism | DTDD | RMET | No correlation found with RMET scores. |
| Mota et al. (2019)^[50]^ | S1: 256 students and community members (23 % male); S2: 346 students and community members (25 % male) | trait narcissism | NARQ; NPI | RMET, MSCEIT, MET, MASC, ERI which assesses emotional recognition with two subtests that comprise different modalities. On the first subtest, facial stimuli from the Pictures of Facial Affect^[70]^ | NPI related to insignificantly lower RMET scores in S1; and significantly worse on MSCEIT in S2 (NARQ significantly worse on MSCEIT and MET and MASC); overestimation of own abilities. |
| Puthillam et al. (2021)^[51]^ | 212 community members (29% male) | trait narcissism | SD3 | GERT-S | NP traits had a significant negative relation with emotion recognition. However, this relation did not hold when controlling for age, nationality, and social desirability. NP traits related to poorer emotion recognition from female faces. |
| Kajonius & Björkman (2020)^[52]^ | 278 community members (27% male) | trait narcissism | SD3 | MET | NP traits had a significant positive correlation with emotion recognition. |
| Schmitt et al. (2020)^[53]^ | 421 students and community members (44% male) | trait narcissism | SD3 | RMET (for Asian and Caucasian eyes) | No correlation found with RMET scores. |
| Raley & Foster (2021)^[54]^ | 291 students (27% male) | trait narcissism | FFNI | Reading the Mind in the Eyes Test (RMET) and GERT | Negative association between NP traits and emotion recognition. |

**Table S1.** Studies on the relation between grandiose narcissism and emotion recognition. DTDD = Dark Triad Dirty Dozen^[66]^; EI = emotional intelligence; ERI - Emotion Recognition Index^[69]^; ERT = Emotion Recognition Task^[67]^; FAB = Florida Affect Battery^[59]^; FFNI – Five Factor Narcissism Inventory^[72]^; GERT = Geneva Emotion Recognition Test^[73]^; GERT-S = Geneva Emotion Recognition Test - Short Version^[71]^; IPT-15 = Interpersonal Perception Task-15^[56],[57]^; MSCEIT = Mayer–Salovey–Caruso Emotional Intelligence Test^[65]^; MASC = Movie for Assessment of Social Cognition^[68]^; MET = Multidimensional Empathy Test^[16]^; NARQ = Narcissistic Admiration and Rivalry Questionnaire^[1]^; NI = Narcissism Inventory^[58]^; NP = narcissistic personality; NPI = Narcissistic Personality Inventory; NPI-16 =16-item version of NPI^[55]^; PNI = Pathological Narcissism Inventory^[61]^; RMET = The Reading the Mind in the Eyes Test^[62]^; SAM = Self-Assessment Manikin^[60]^; S1= Study 1; S2 = Study 2; SD3 = Short Dark Triad Scale^[20]^; UCDSEE = University of California, Davis, Set of Emotion Expressions^[63]^;

|  | **Signal Model Agentic Narcissism** | | | | | **Signal Model Communal Narcissism** | | | | |
| --- | --- | --- | --- | --- | --- | --- | --- | --- | --- | --- |
| *Predictors* | *Estimates* | *SE* | *95% CI* | *p* | *df* | *Estimates* | *SE* | *95% CI* | *p* | *df* |
| (Intercept) | 4.96 | 0.06 | 4.84 – 5.08 | **< .001** | 144.68 | 4.96 | 0.06 | 4.84 – 5.08 | **< .001** | 144.96 |
| Agentic Narcissism | 0.01 | 0.02 | -0.03 – 0.05 | **<** .646 | 145.02 |  |  |  |  |  |
| Communal Narcissism |  |  |  |  |  | 0.17 | 0.05 | 0.07 – 0.27 | **<.002** | 145.01 |
| Disgust - Happiness | -0.89 | 0.08 | -1.06 – -0.73 | **< .001** | 146.00 | -0.89 | 0.08 | -1.06 – -0.73 | **< .001** | 146.00 |
| Anger – Disgust | -0.23 | 0.08 | -0.39 – -0.07 | **< .004** | 145.96 | -0.23 | 0.08 | -0.39 – -0.07 | **< .004** | 145.95 |
| Sadness – Anger | -0.48 | 0.08 | -0.64 – -0.32 | **< .001** | 146.01 | -0.48 | 0.08 | -0.64 – -0.32 | **< .001** | 146.00 |
| ICC | 0.30 |  |  |  |  | 0.29 |  |  |  |  |
| N | 147_id_ |  |  |  |  | 147_id_ |  |  |  |  |
| Observations | 7053 |  |  |  |  | 7053 |  |  |  |  |
| Marginal R^2^/Conditional R^2^ | 0.115/0.384 |  |  |  |  | 0.125/0.382 |  |  |  |  |

**Table S2.** Results of mixed effect models in Study 1: Signal*.* ICC = intraclass correlation coefficient.

|  | **Noise Model Agentic Narcissism** | | | | | **Noise Model Communal Narcissism** | | | | |
| --- | --- | --- | --- | --- | --- | --- | --- | --- | --- | --- |
| *Predictors* | *Estimates* | *SE* | *95% CI* | *p* | *df* | *Estimates* | *SE* | *95% CI* | *p* | *df* |
| (Intercept) | 2.32 | 0.04 | 2.24 – 2.40 | **< .001** | 144.26 | 2.32 | 0.04 | 2.25 – 2.40 | **< .001** | 143.89 |
| Agentic Narcissism | 0.03 | 0.01 | 0.01 – 0.05 | **< .010** | 136.88 |  |  |  |  |  |
| Communal Narcissism |  |  |  |  |  | 0.10 | 0.03 | 0.05 – 0.16 | **< .001** | 137.10 |
| Anger – Happiness | 0.40 | 0.04 | 0.33– 0.47 | **< .001** | 106.28 | 0.40 | 0.04 | 0.33 – 0.47 | **< .001** | 107.64 |
| Disgust – Anger | 0.04 | 0.02 | -0.01 – 0.08 | **<** .083 | 68.34 | 0.04 | 0.02 | -0.01 – 0.08 | **<** .083 | 63.32 |
| Sadness – Disgust | 0.09 | 0.02 | 0.05 – 0.13 | **< .001** | 210.90 | 0.09 | 0.02 | 0.05 – 0.13 | **< .001** | 209.15 |
| ICC | 0.56 |  |  |  |  | 0.55 |  |  |  |  |
| N | 147_id_ |  |  |  |  | 147_id_ |  |  |  |  |
| Observations | 7053 |  |  |  |  | 7053 |  |  |  |  |
| Marginal R^2^/Conditional R^2^ | 0.097/0.602 |  |  |  |  | 0.109/0.597 |  |  |  |  |

**Table S3.** Results of mixed effect models in Study 1: Noise ICC = intraclass correlation coefficient.

|  | **Signal Model Agentic Narcissism** | | | | | **Signal Model Communal Narcissism** | | | | |
| --- | --- | --- | --- | --- | --- | --- | --- | --- | --- | --- |
| *Predictors* | *Estimates* | *SE* | *95% CI* | *p* | *df* | *Estimates* | *SE* | *95% CI* | *p* | *df* |
| (Intercept) | 4.87 | 0.03 | 4.81 – 4.94 | **< .001** | 517.23 | 4.87 | 0.03 | 4.79 – 4.93 | **< .001** | 517.64 |
| Agentic Narcissism | -0.05 | 0.03 | -0.11 – 0.001 | **<** .063 | 517.99 |  |  |  |  |  |
| Communal Narcissism |  |  |  |  |  | -0.04 | 0.03 | -0.10 – 0.02 | **<** .165 | 518.43 |
| Neutral – Surprise | -0.58 | 0.06 | -0.70 – -0.47 | **< .001** | 880.81 | -0.58 | 0.06 | -0.70 – -0.47 | **< .001** | 881.29 |
| Disgust – Neutral | -0.42 | 0.07 | -0.56 – -0.28 | **< .001** | 521.50 | -0.42 | 0.07 | -0.56 – -0.28 | **< .001** | 521.58 |
| Happiness – Disgust | -0.46 | 0.06 | -0.59 – -0.34 | **< .001** | 525.46 | -0.46 | 0.06 | -0.59 – -0.34 | **< .001** | 525.48 |
| Anger – Happiness | -0.04 | 0.06 | -0.16 – -0.08 | **<** .526 | 526.44 | -0.04 | 0.06 | -0.16 – -0.08 | **<** .526 | 526.58 |
| Sadness – Anger | -0.79 | 0.07 | -0.93 – -0.65 | **< .001** | 516.10 | -0.79 | 0.07 | -0.93 – -0.65 | **< .001** | 516.09 |
| Fear - Sadness | -0.03 | 0.07 | -0.17 – -0.12 | **<** .716 | 520.11 | -0.03 | 0.07 | -0.17 – -0.12 | **<** .715 | 520.03 |
| ICC | 0.23 |  |  |  |  | 0.23 |  |  |  |  |
| N | 521_id_ |  |  |  |  | 521_id_ |  |  |  |  |
| Observations | 14496 |  |  |  |  | 14496 |  |  |  |  |
| Marginal R^2^/Conditional R^2^ | 0.142/0.343 |  |  |  |  | 0.141/0.343 |  |  |  |  |

**Table S4.** Results of mixed effect models in Study 2: Signal. ICC = intraclass correlation coefficient.

|  | **Noise Model Agentic Narcissism** | | | | | **Noise Model Communal Narcissism** | | | | |
| --- | --- | --- | --- | --- | --- | --- | --- | --- | --- | --- |
| *Predictors* | *Estimates* | *SE* | *95% CI* | *p* | *df* | *Estimates* | *SE* | *95% CI* | *p* | *df* |
| (Intercept) | 2.04 | 0.03 | 1.98 – 2.10 | **< .001** | 464.42 | 2.04 | 0.03 | 1.98 – 2.10 | **< .001** | 435.90 |
| Agentic Narcissism | 0.17 | 0.03 | 0.11 – 0.22 | **< .001** | 555.67 |  |  |  |  |  |
| Communal Narcissism |  |  |  |  |  | 0.16 | 0.03 | 0.11 – 0.21 | **< .001** | 560.20 |
| Neutral – Surprise | 0.02 | 0.02 | -0.01 – 0.06 | **<** .239 | 614.58 | 0.02 | 0.02 | -0.01 – 0.06 | **<** .230 | 543.07 |
| Happiness – Neutral | 0.01 | 0.02 | -0.02 – 0.05 | **<** .443 | 194.26 | 0.01 | 0.02 | -0.02 – 0.05 | **<** .444 | 135.67 |
| Disgust – Happiness | 0.26 | 0.02 | 0.21 – 0.30 | **< .001** | 234.54 | 0.26 | 0.02 | 0.21 – 0.30 | **< .001** | 138.70 |
| Anger – Disgust | 0.18 | 0.02 | 0.14 – 0.23 | **< .001** | 368.50 | 0.18 | 0.02 | 0.14 – 0.23 | **< .001** | 339.05 |
| Sadness – Anger | 0.07 | 0.02 | 0.04 – 0.11 | **< .001** | 453.05 | 0.07 | 0.02 | 0.04 – 0.11 | **< .001** | 426.75 |
| Fear - Sadness | 0.08 | 0.02 | 0.04 – 0.11 | **< .001** | 469.04 | 0.08 | 0.02 | 0.04 – 0.11 | **< .001** | 345.87 |
| ICC | 0.67 |  |  |  |  | 0.67 |  |  |  |  |
| N | 521_id_ |  |  |  |  | 521_id_ |  |  |  |  |
| Observations | 14496 |  |  |  |  | 14496 |  |  |  |  |
| Marginal R^2^/Conditional R^2^ | 0.113/0.708 |  |  |  |  | 0.109/0.708 |  |  |  |  |

**Table S5.** Results of mixed effect models in Study 2: Noise. ICC = intraclass correlation coefficient.
